# Supplementary material for: Uniform thin ice on ultraflat graphene for high-resolution cryo-EM
Source: Nat Methods. 2022 Dec 15;20(1):123–30. doi: 10.1038/s41592-022-01693-y (PMC9834055; doi:10.1038/s41592-022-01693-y)
Supplement: Supplementary file 1 — Supplementary Protocols, Figs. 1–8 and Tables 1 and 2. [file 41592_2022_1693_MOESM1_ESM.pdf]

nature methods

Article

https://doi.org/10.1038/s41592-022-01693-y

Uniform thin ice on ultraflat graphene for high-resolution cryo-EM

In the format provided by the authors and unedited

Contents

Supplementary Protocol 2
Supplementary Fig. 1 5
Supplementary Fig. 2 6
Supplementary Fig. 3 7
Supplementary Fig. 4 8
Supplementary Fig. 5 9
Supplementary Fig. 6 10
Supplementary Fig. 7 10
Supplementary Fig. 8 11
Supplementary Table 1 12
Supplementary Table 2 13
Supplementary References 14

Supplementary Protocol

Production of wafer-scale Cu(111)/sapphire substrate

1. The single-side polished C-plane (0001) sapphire wafers were purchased from Jiangsu HELIOS, China. The sapphire wafer exhibited an ultraflat surface with an average roughness of <0.2 nm and a miscut angle of <0.5°.
2. Before Cu film deposition, the sapphire wafers were annealed at 1000 °C for 6 h in an oxygen atmosphere.
3. Then, a 500-nm-thick Cu film was deposited onto sapphire wafer by magnetron sputtering within 30 min using a PVD equipment (Sputter film, SF2), whose radio frequency power was ~300 W with a basal pressure of ~0.15 Pa.
4. Subsequently, the wafers were annealed in a tube furnace at 1000 °C with 500 sccm Ar and 10 sccm H_2 at atmospheric pressure for one hour to acquire the Cu(111) thin film on sapphire substrate.

CVD growth of ultraflat graphene (UFG) on Cu(111)/sapphire

5. The Cu(111)/sapphire wafer was placed on the surface of the other sapphire wafer (Supplementary Fig. 7) in a tube furnace.
6. To synthesize the 4-inch UFG, the Cu(111) wafer was heated to 1000 °C within 60 min under a flow of 2000 sccm Ar and 40 sccm H_2 at atmospheric pressure.
7. 40 sccm CH_4 (0.1% diluted in Ar) was then introduced for graphene growth. Usually, it took two hours for graphene to cover the surface of Cu(111) wafer fully.
8. CH_4 gas flow was shut down as soon as the growth was completed, and the sample was then cooled to room temperature.

CVD growth of graphene on copper foil

9. Commercially available Cu foil (Alfa Aesar #46986 or #46365) was used to grow graphene film. The Cu foil was first electrochemically polished in phosphoric acid and ethylene glycol solution (v/v = 3:1) with a voltage of 2.4 V for 12 min.
10. The polished copper foil was rinsed with deionized water and ethanol in sequence to remove the electrolyte. A nitrogen gun can be used to facilitate the drying process of polished copper foil.
11. Then the copper foil was annealed at 1000 °C for 0.5 h in a tube furnace under the flow of 100 sccm H_2 with a corresponding pressure of ~100 Pa.
12. Subsequently, 1 sccm CH_4 was introduced into the tube furnace for graphene growth. After one hour, graphene film was synthesized on the copper foil.
13. Finally, the graphene/copper foil was pulled out quickly from the heating area of tube furnace and cooled down to the room temperature under the flow of H_2.

Face-to-face transfer of graphene onto TEM grids

14. The commercial Au holey carbon grids (Quantifoil, Au-300 or 200 mesh-R1.2/1.3) were used to prepare graphene grids. Note that the grids with copper bars should be avoided because the copper bars can be etched by the $(NH_4)_2S_2O_8$ aqueous solution.

15. Face-to-face transfer: To transfer UFG from Cu(111)/sapphire wafer to TEM grids, we placed a batch of TEM grids on the wafer. Then we dropped a few droplets of isopropanol to cover the surface of these TEM grids. After the solution was volatilized, TEM grids and UFG were combined (as shown in Supplementary Fig. 8).

    The same treatment was performed to combine the TEM grids and rough graphene on the copper foil.

16. Etching: The grids/graphene/Cu(111)/sapphire composite was submerged into 1 M $(NH_4)_2S_2O_8$ aqueous solution to etch the Cu(111) film away. After the copper film was etched, the graphene grids were obtained with UFG covered on the holey carbon film.

    As for the etching of copper foil, the grids/graphene/copper foil composite was floated on the surface of $(NH_4)_2S_2O_8$ solution because the copper foil was light enough to be supported by the surface tension of solution.

17. Rinsing: Graphene grids were submerged into deionized water to wash away the inorganic salts. Then they were transferred into isopropanol for further rinsing.

18. Drying: The graphene grids were normally dried in the super-clean room to avoid extra contaminants after being taken out from the isopropyl alcohol.

Glow discharging of graphene grids

19. To render the hydrophilic graphene grids, graphene grids were glow-discharged for ~12 s at the low mode in a plasma cleaner (Harrick, PDC-32G) after the chamber was evacuated for 2 min.

Cryo-EM specimen preparation

20. Streptavidin was purchased from New England Biolabs (Catalog: N7021S) and hemoglobin was purchased from Sigma-Aldrich (Catalog: H7379).
21. For 20S proteasome purification, we firstly constructed a plasma containing the α and β subunits of 20S proteasome, and the N terminal of β subunit was His-tagged. The plasma was then transformed into *Escherichia coli* competent cells and allowed for overnight expression. The cells were centrifuged and sonicated to obtain the cell lysates, which were finally loaded onto nickel column (GE Healthcare) for affinity purification[1].
22. For alpha-fetoprotein (AFP), we firstly transfected the recombinant vector of pCAG-*afp*-His-Strep into HEK-293F cells. After transfection, the cells were cultured for 2 days and then harvested by centrifugation. The pellets were resuspended in lysis buffer and loaded onto Strep-Tactin XT for affinity purification. After that, we further performed gel filtration to get the purified AFP sample[2].

23. A 3-μL drop of a sample solution containing 0.2 μM 20S proteasome or 0.1 μM streptavidin or 6 mg/mL hemoglobin or 1 μM human alpha-fetoprotein was pipetted onto graphene grids, which had been previously glow-discharged.
24. The grids were transferred into an FEI Vitrobot with humidity of 100% and temperature of 8 °C and blotted for 1s with the force of -2.
25. Afterwards, the grid was plunge-frozen into liquid ethane and kept in liquid nitrogen for further cryo-EM imaging.

Cryo-EM data collection and processing

26. We used AutoEMation Software[3] written by Dr. Jianlin Lei in Tsinghua University to automatically collect single-particle datasets on an FEI Titan Krios (300 kV), equipped with an energy filter and a Gatan K3 summit detector, with an accumulated dose of 50 $e^-/Å^2$. All the movies contained 32 frames, which were motion-corrected by MotionCor2 algorithm[4]. The CTF values were estimated by CTFFIND4[5].
27. Then we used Relion3.1[6] to pick the particles and performed further iteratively 2D classification to discard bad particles. The remaining good particles were imported for 3D classification and refinement. For hemoglobin, C2 symmetry was imposed in the 3D refinement with the final particle number of 105,000. To generate the atomic model of hemoglobin, the previously published coordinate (PDB: 7VDE)[7] was docked into the cryo-EM map and real-space refined in PHENIX[8]. For alpha-fetoprotein, we used 354,264 particles in the final 3D reconstruction to obtain a 2.6-Å density map. For streptavidin, D2 symmetry was applied in the 3D refinement step with the final particle number of 260,390. The resolution was determined by the Fourier Shell Correlation (FSC)=0.143 cutoff criteria.
28. We performed CTF Refinement in Relion3.0 to estimate the local defocus of particles in individual micrographs and plotted the defocus range.
29. To measure the particle motion on graphene support, we performed Bayesian polishing to determine the particles coordinates in each frame. These coordinates were then used to calculate the root-mean-squared displacements of each particle and plotted versus dose. We carried out three particle-motion measurements of both UFG and rough graphene (RG), every of which was based on thousands of particles.

Cryo-electron tomography analysis

30. Cryo-ET tilt series were acquired by SerialEM software[9] from +60° to -60° with a step of 3°, on an FEI Titan Krios microscope (300 kV), equipped with a Gatan K2 camera. At each tilt angle, we collected movies containing 8 frames, with a sum dose of ~3 $e^-/Å^2/s$, and the total dose for every tilt series (+60° to -60°) was 120 $e^-/Å^2$. The pixel size was 1.25 Å.
31. The movies were firstly motion-corrected by MotionCor2[4] and then imported into Etomo[10] for alignment and reconstruction. The position of protein particles within the ice was manually identified and plotted[1].

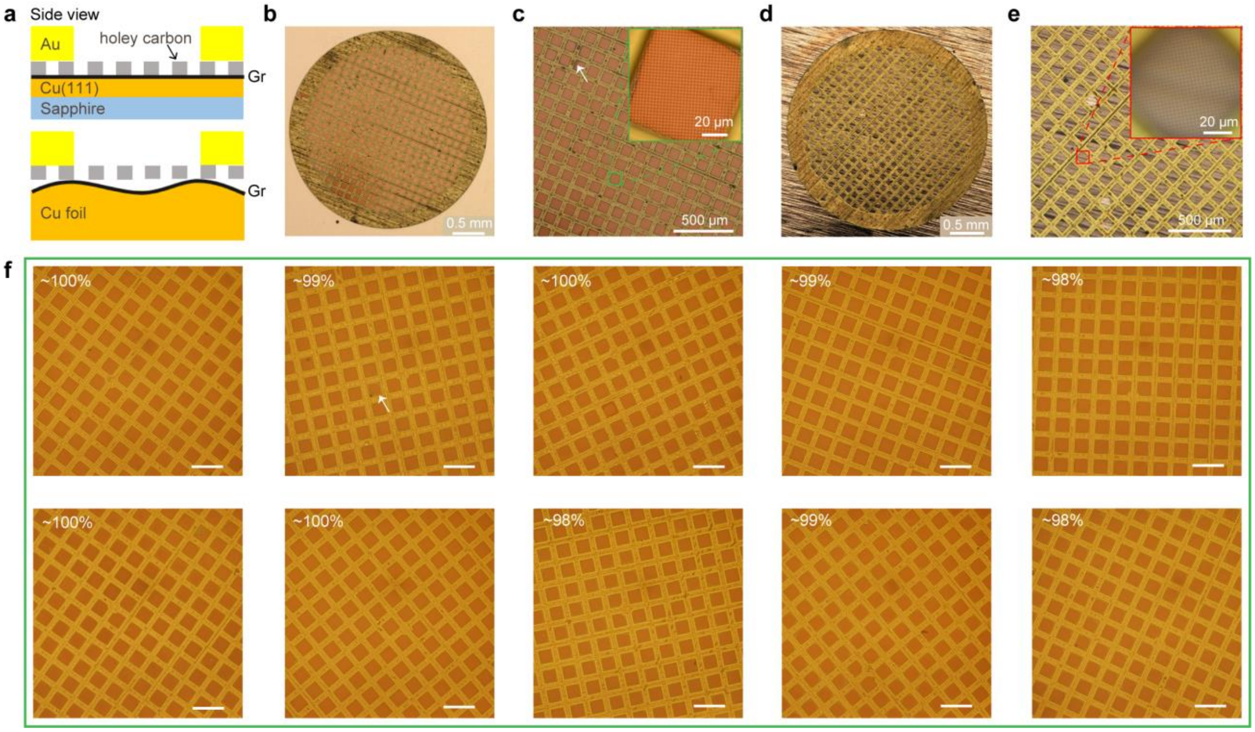

Supplementary Fig. 1 | Surface roughness dominating the interfacial contact between graphene and EM grids during the transfer procedure. a, Schematic side views showing the EM grids attached to the UFG (top) and RG (down), respectively. The grid was well attached to the UFG surface, while some regions of grid were detached from the RG on the copper foil. **b**, Typical OM image of EM grid on the UFG wafer. **c**, The high-magnification OM image showing the holey carbon film of grid was tightly attached to the UFG. Only the grey region as the white arrow labelled was detached from the graphene surface. **d**, Typical OM image of EM grid on the RG film. **e**, The high-magnification OM image revealing that much more regions of the holey carbon film of the grid were detached from the RG, as evidenced by the different focal heights of holey carbon and graphene surface. **f**, Typical OM images of grids showing a universally well contact between EM grid and UFG (>98%). Scale bars, 200 μm.

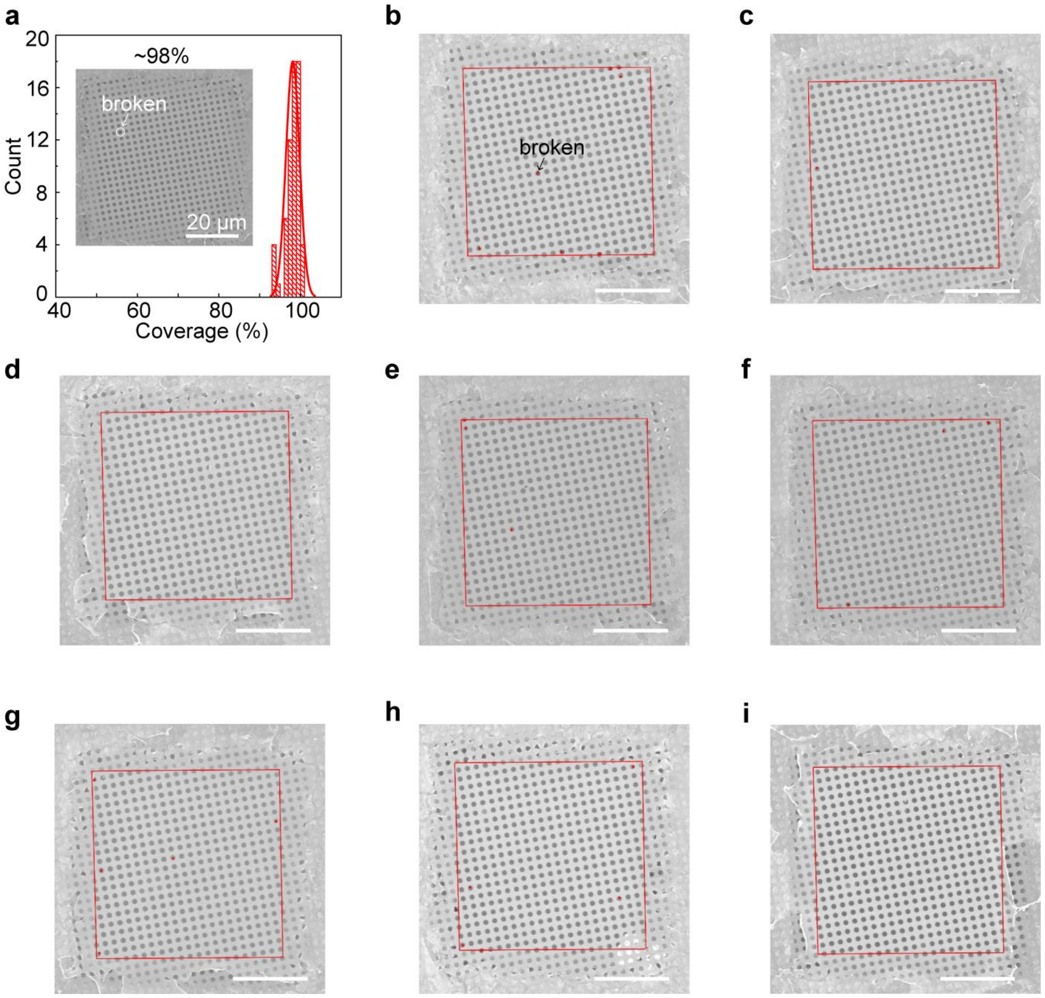

Supplementary Fig. 2 | High-coverage suspended UFG membranes on EM grids. a, Coverage distribution of suspended graphene membranes on an EM grid. The average coverage of suspended graphene was ~98%. Inset: representative SEM image of suspended graphene membranes on the grid. **b-i,** Typical SEM images showing a universally high coverage of suspended graphene. Scale bars, 20 µm.

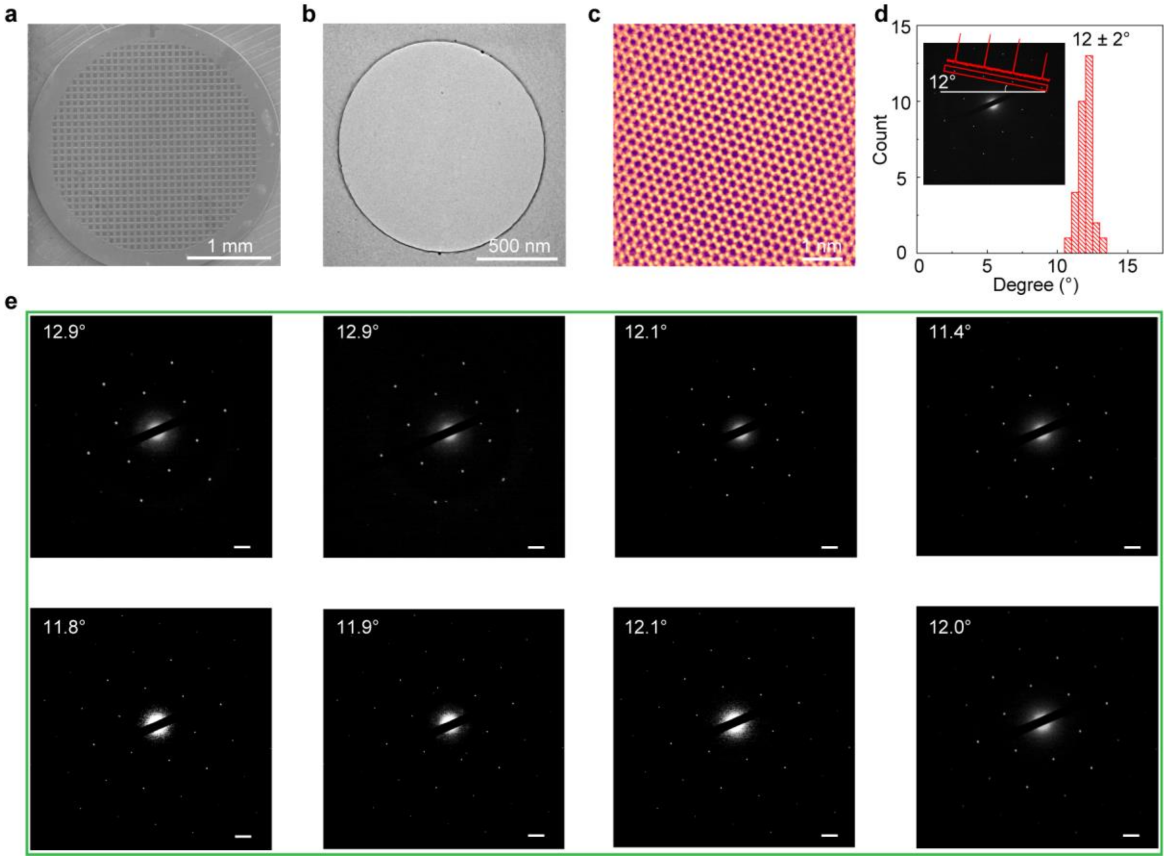

Supplementary Fig. 3 | Ultraclean and single-crystal graphene grid. a, SEM image of a graphene grid. **b,** Representative TEM image showing the ultraclean surface of suspended graphene on a hole of grid. **c,** Typical atomic-resolution STEM-HAADF image of graphene lattice. No transfer-induced residues were observed on the graphene surface. **d,** Angle distribution extracted from extensive selected area electron diffraction (SAED) patterns within the graphene grid in (**a**). The narrow angle distribution (12±2°) showed that the graphene film was a single crystal. Inset: representative SAED pattern of suspended graphene and corresponding intensity profile of the diffraction patterns in the red box. The 12° was the measured angle between the horizontal line and diffraction patterns in the box. **e,** Typical SAED patterns collected from the single-crystal graphene grid in (**a**). Scale bars, 2 nm^{-1}.

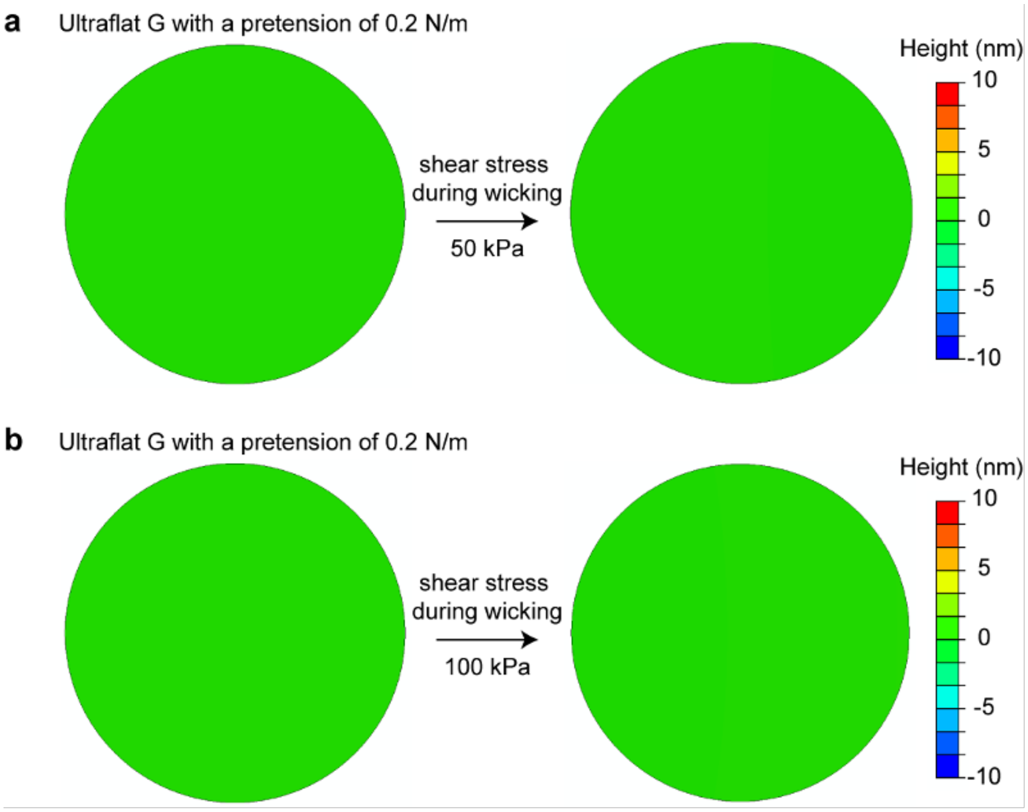

Supplementary Fig. 4 | Finite element simulations of pretensioned UFG under different shear stresses. a-b, Pretensioned UFG maintains its flatness under the shear stress of ~50 kPa (**a**) and ~100 kPa (**b**), respectively. Diameters of suspended graphene: 1.2 µm.

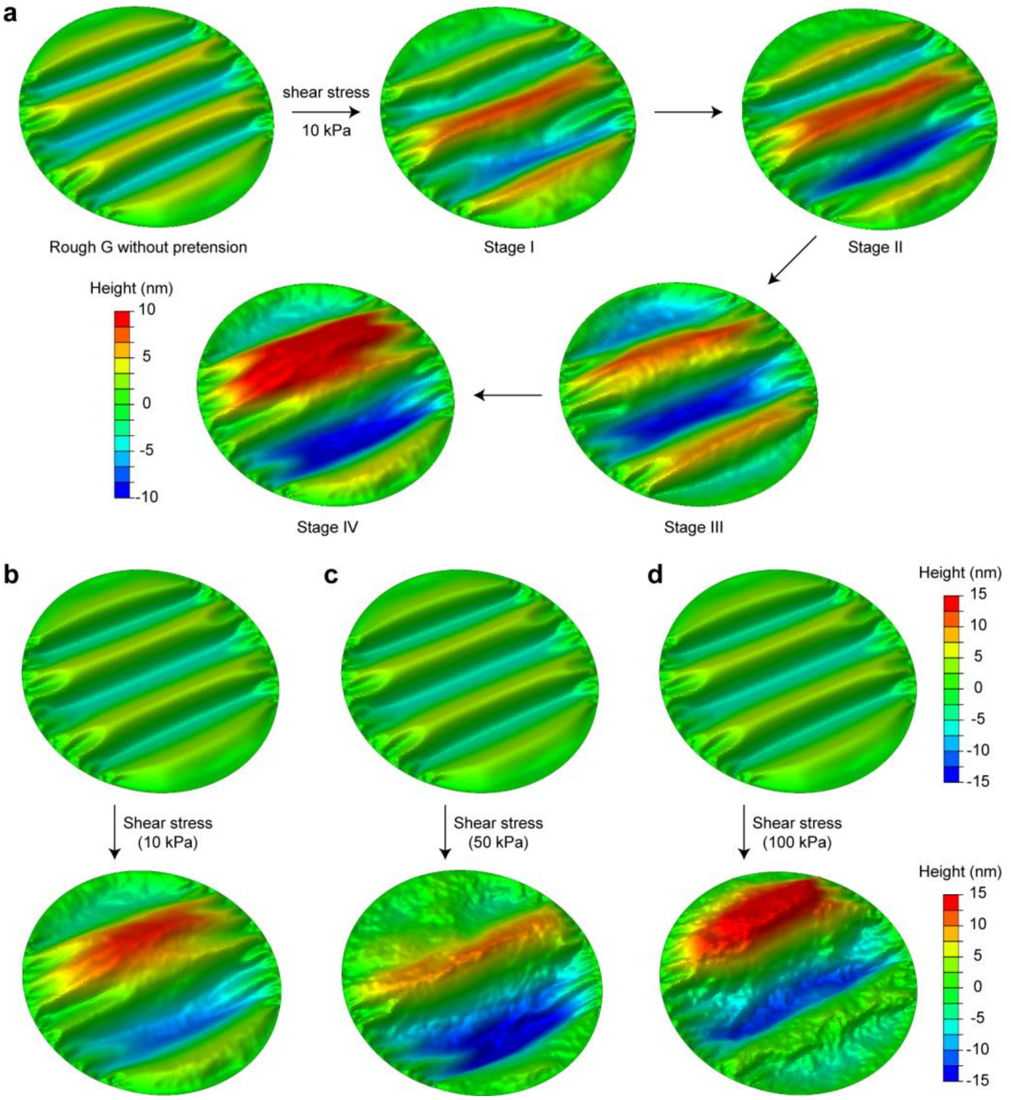

Supplementary Fig. 5 | Finite element simulations of RG under different shear stresses. a, Deformation process of suspended RG under the shear stress of ~10 kPa. The adjacent wrinkles merged, and the amplitude of the resultant wrinkle increased. **b-d,** Structural distortions of RG membranes under the shear stress of ~10 kPa (**b**), ~50 kPa (**c**), and ~100 kPa (**d**), respectively. The height variations of RG were remarkably enlarged with increased shear stress, and the maximum height difference of RG can be up to 20~30 nm. Diameters of suspended graphene: 1.2 μm.

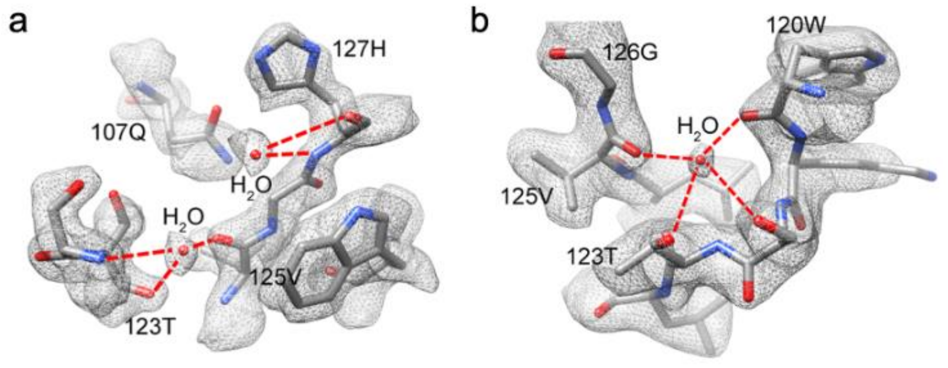

Supplementary Fig. 6 | Selected density regions of 52-kDa streptavidin with the corresponding atomic models docked. The H_2O molecules and side chain of residues were clearly observed. Hydrogen bonds were indicated by red dash lines.

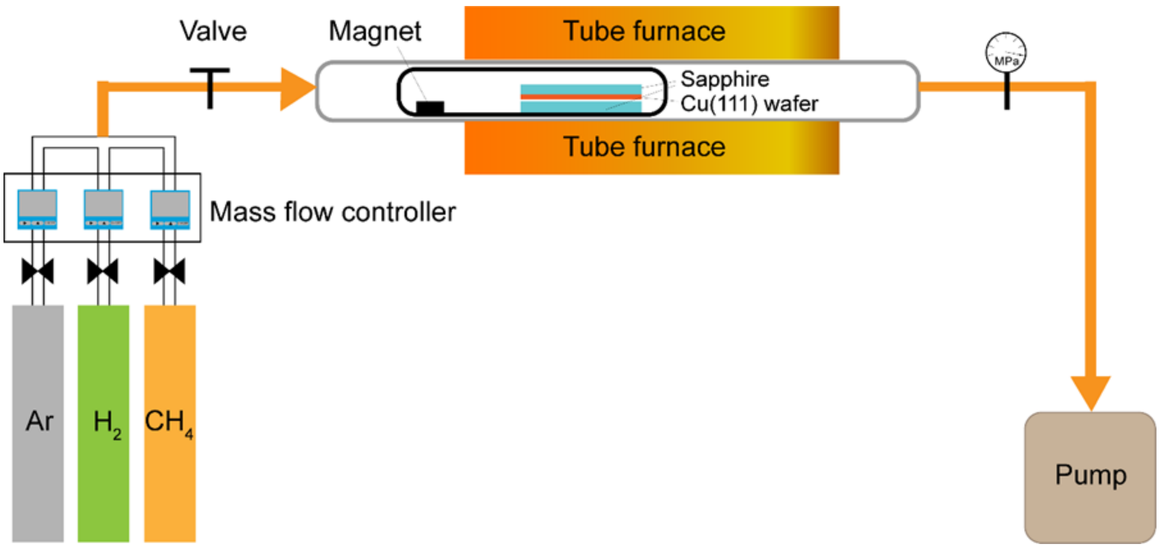

Supplementary Fig. 7 | Make and model of the CVD system. The home-made CVD system typically consists of gas supply system (high-purity compressed gas, JINGHUI GAS), control system (mass flow controller, S48 32/HMT, HORIBAR METRON), CVD reactor (tube furnace, Thermo SCIENTIFIC; Quartz tube, 120×1500 mm) and pump system (pump, GLD-N051, ULVAC Inc).

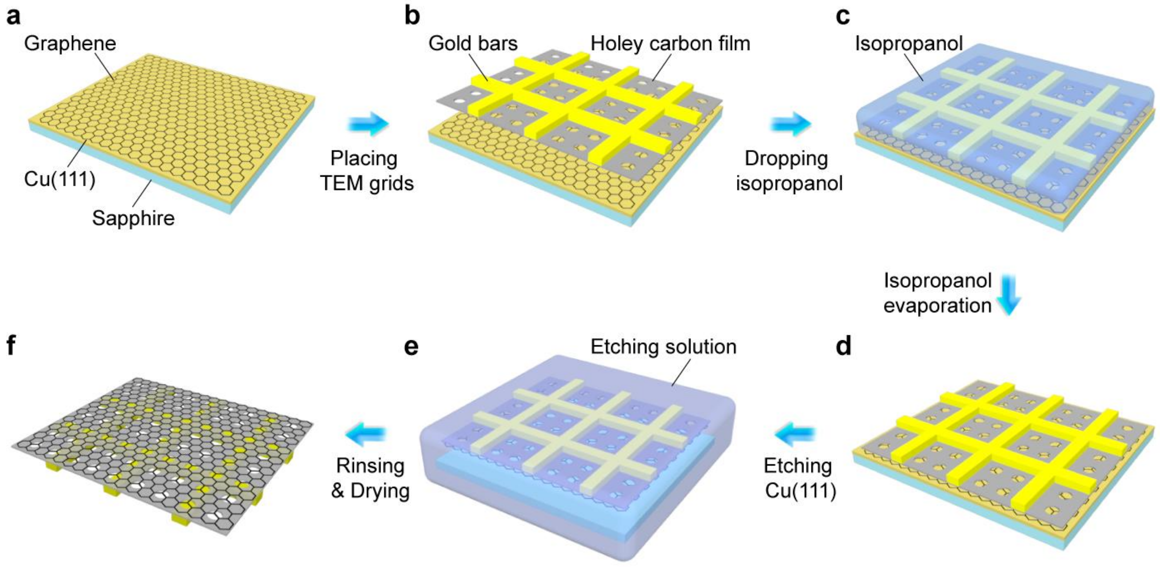

Supplementary Fig. 8 | Schematic illustration of face-to-face transfer method. a, UFG film on the Cu(111)/sapphire substrate. **b,** Placing TEM grids onto the UFG/Cu(111)/sapphire. **c,** Dropping isopropanol solution to cover the surface of TEM grids on graphene/Cu(111)/sapphire. **d,** Combined TEM grids/graphene/Cu(111)/sapphire composite after isopropanol evaporation. **e,** Submerged composite in the etching solution to etch the Cu(111) film off. **f,** Graphene-coated TEM grids after being rinsed and dried.

Supplementary Table 1 | Summary of Young's modulus and mechanical strength of graphene reported in literature.

Graphene	Young's modulus (GPa)	Mechanical strength (GPa)	Hole size (μm)	Reference
Exfoliated	1010 ± 150	130 ± 10	1-1.5	*Science* **2008**, *321*, 385
Exfoliated	897 ± 41	83.6–104.5	0.5-3	*Nat. Phys.* **2015**, *11*, 26
Exfoliated	1060 ± 80	---	1-1.5	*ACS Nano* **2016**, *10*, 1820
CVD	161 ± 147	35	2	*Nano Lett.* **2011**, *11*, 2259
Large-grain CVD Graphene	1010 ± 50	103	1-1.5	*Science* **2013**, *340, 1073*
Small-grain CVD Graphene	980 ± 50	98.5		
CVD	540 ± 33	13.6	2.2	*ACS Nano* **2013**, *7*, 1171
CVD	423	28.7	2.2	*ACS Nano* **2014**, *8*, 10246
Rough Graphene (RG)	721.5 ± 139.4	93.1±24.9	1.2	This work
Ultraflat Graphene (UFG)	933.1 ± 171.0	145.0±13.3		

Supplementary Table 2 | Summary of cryo-EM data collection and processing

	20S proteasome	20S proteasome	Hemoglobin	Alpha-fetoprotein	Streptavidin
Molecular weight (kDa)	700	700	64	67	52
Supporting film	Rough Graphene (RG)	Ultraflat Graphene (UFG)	Ultraflat Graphene (UFG)	Ultraflat Graphene (UFG)	Ultraflat Graphene (UFG)
Magnification	29,000	29,000	165,000	165,000	165,000
Voltage (kV)	300	300	300	300	300
Pixel size (Å)	0.97	0.97	0.5191	0.5191	0.5191
Electron exposure ($e–/Å^2$)	50	50	50	50	50
Defocus range (μm)	0.6-2.5	0.5-2.3	0.6-2.4	0.6-2.5	0.7-2.2
Symmetry imposed	D7	D7	C2	C1	D2
Micrographs （no.）	324	316	5,604	2,290	2,332
Initial particle images (no.)	150,774	143,954	5,761,072	1,660,932	3,134,598
Final particle. images (no.)	12,978	7,152	105,000	354,264	260,390
Resolution (Å)	3.0	2.8	3.5	2.6	2.2

Supplementary References

1 Liu, N. *et al.* Bioactive Functionalized Monolayer Graphene for High-Resolution Cryo-Electron Microscopy. *J. Am. Chem. Soc.* **141**, 4016-4025, (2019).

2 Lin, B. *et al.* Purification and characterization of a bioactive alpha-fetoprotein produced by HEK-293 cells. *Protein Expres. Purif.* **136**, 1-6 (2017).

3 Lei, J. & Frank, J. Automated acquisition of cryo-electron micrographs for single particle reconstruction on an FEI Tecnai electron microscope. *J. Struct. Biol.* **150**, 69-80, (2005).

4 Zheng, S. Q. *et al.* MotionCor2: anisotropic correction of beam-induced motion for improved cryo-electron microscopy. *Nat. Methods* **14**, 331-332 (2017).

5 Rohou, A. & Grigorieff, N. CTFFIND4: Fast and accurate defocus estimation from electron micrographs. *J. Struct. Biol.* **192**, 216-221 (2015).

6 Scheres, S. H. RELION: implementation of a Bayesian approach to cryo-EM structure determination. *J. Struct. Biol.* **180**, 519-530 (2012).

7 Fan, H. C. *et al.* A cryo-electron microscopy support film formed by 2D crystals of hydrophobin HFBI. *Nat. Commun.* **12**, 7257 (2021).

8 Adams, P. D. *et al.* PHENIX: a comprehensive Python-based system for macromolecular structure solution. *Acta. Crystallogr.* D **66**, 213-221(2010).

9 Mastronarde, D. N. Automated electron microscope tomography using robust prediction of specimen movements. *J. Struct. Biol.* **152**, 36-51 (2005).

10 Kremer, J. R., Mastronarde, D. N. & McIntosh, J. R. Computer visualization of three-dimensional image data using IMOD. *J. Struct. Biol.* **116**, 71-76 (1996).
