# Supplementary material for: Uniform thin ice on ultraflat graphene for high-resolution cryo-EM
Source: Nat Methods. 2022 Dec 15;20(1):123–30. doi: 10.1038/s41592-022-01693-y (PMC9834055; doi:10.1038/s41592-022-01693-y)
Supplement: Supplementary file 2 — Reporting Summary [file 41592_2022_1693_MOESM2_ESM.pdf]

nature research

Corresponding author(s): Hong-Wei Wang

Last updated by author(s): Sep 28, 2022

Reporting Summary

Nature Research wishes to improve the reproducibility of the work that we publish. This form provides structure for consistency and transparency in reporting. For further information on Nature Research policies, see our Editorial Policies and the Editorial Policy Checklist.

Statistics

For all statistical analyses, confirm that the following items are present in the figure legend, table legend, main text, or Methods section.

n/a	Confirmed	
☐	☒	The exact sample size (n) for each experimental group/condition, given as a discrete number and unit of measurement
☐	☒	A statement on whether measurements were taken from distinct samples or whether the same sample was measured repeatedly
☒	☐	The statistical test(s) used AND whether they are one- or two-sided<br>*Only common tests should be described solely by name; describe more complex techniques in the Methods section.*
☒	☐	A description of all covariates tested
☒	☐	A description of any assumptions or corrections, such as tests of normality and adjustment for multiple comparisons
☐	☒	A full description of the statistical parameters including central tendency (e.g. means) or other basic estimates (e.g. regression coefficient) AND variation (e.g. standard deviation) or associated estimates of uncertainty (e.g. confidence intervals)
☒	☐	For null hypothesis testing, the test statistic (e.g. F, t, r) with confidence intervals, effect sizes, degrees of freedom and P value noted<br>*Give P values as exact values whenever suitable.*
☒	☐	For Bayesian analysis, information on the choice of priors and Markov chain Monte Carlo settings
☒	☐	For hierarchical and complex designs, identification of the appropriate level for tests and full reporting of outcomes
☒	☐	Estimates of effect sizes (e.g. Cohen's d, Pearson's r), indicating how they were calculated

Our web collection on statistics for biologists contains articles on many of the points above.

Software and code

Policy information about availability of computer code

Data collection	We used ABAQUS 2017 software to perform finite element simulations of graphene rippling. We used AutoEMation software to collect single-particle cryo-EM datasets, written by Dr. Jianlin Lei at Tsinghua University. We used SerialEM (version 3.8) software to collect tilt series.
Data analysis	We used MotionCor2 (version 1.1.0) to correct the beam-induced motion of cryo-EM micrographs and used Relion (version 3.1.3) to perform 3D reconstruction. The CTF values of these motion-corrected micrographs were determined by CTFFIND4 algorithm (version 4.15). The structural analysis was performed in UCSF Chimera (version 1.13.1). The coordinate was generated in PHENIX (version1.14-3260). For cryo-ET reconstruction, we used Etomo (version 4.11) to align and reconstruct the tomograms. All these softwares are open-source. All these softwares are open-source.

For manuscripts utilizing custom algorithms or software that are central to the research but not yet described in published literature, software must be made available to editors and reviewers. We strongly encourage code deposition in a community repository (e.g. GitHub). See the Nature Research guidelines for submitting code & software for further information.

Data

Policy information about availability of data

All manuscripts must include a data availability statement. This statement should provide the following information, where applicable:

- Accession codes, unique identifiers, or web links for publicly available datasets
- A list of figures that have associated raw data
- A description of any restrictions on data availability

Data supporting the findings in this manuscript are available from the corresponding authors upon reasonable requests. The coordinates and density maps of streptavidin, hemoglobin and alpha-fetoprotein have been deposited in the RCSB Protein Data Bank (PDB) under the accession number 8GVK, 7XGY and 7YIM, and the EMDB under accession number EMD-32099, EMD-33189 and EMD-33861, respectively. The raw cryo-EM dataset of streptavidin, containing the unaligned

movies and particles, has been deposited into EMPIAR under accession number EMPIAR-11217. Source data of Fig. 2c, Fig. 3b and c, Fig. 4b, d, f, h, i, j and k, Extended Data Fig. 1c and f, Extended Data Fig. 2e, Extended Data Fig. 3b and e, Extended Data Fig. 4g and h, Extended Data Fig. 5c-f, Extended Data Fig. 8, and Extended Data Fig. 9a-c are provided as Source Data files.

Field-specific reporting

Please select the one below that is the best fit for your research. If you are not sure, read the appropriate sections before making your selection.

☒ Life sciences ☐ Behavioural & social sciences ☐ Ecological, evolutionary & environmental sciences

For a reference copy of the document with all sections, see nature.com/documents/nr-reporting-summary-flat.pdf

Life sciences study design

All studies must disclose on these points even when the disclosure is negative.

Sample size	For cryo-EM analysis, the particle numbers used for the final reconstructions of hemoglobin, alpha-fetoprotein and streptavidin were 105,000, 354,264 and 260,390, respectively, and the reported resolutions were 3.5 Å, 2.6 Å and 2.2 Å, respectively, estimated by the Fourier Shell Correction (FSC)=0.143 cutoff criteria. In cryo-EM field, particle number used for final structural reconstruction varies greatly, which is well acceptable for such sample size used here.<br>To plot and compare the distribution of defocus ranges, we used 313 UFG-supported micrographs and 324 RG-supported micrographs. For cryo-EM reconstruction of 20S proteasome, we normally collect hundreds of micrographs, which are enough for us to get a reconstruction at near-atomic resolution and analyze the defocus ranges.<br>To measure the particle motion, we carried out three particle-motion measurements of both UFG and RG, every of which was based on thousands of particles. Such example size and times of repeated experiments have been widely used to characterize beam-induced motion.
Data exclusions	For cryo-EM reconstruction, particles grouped in bad classes with poorly defined features were excluded, because these particles were normally denatured or dissociated samples, which were harmful for high-resolution 3D reconstruction.
Replication	Particle-motion measurements were repeated 3 times (as indicated in Methods), and all attempts at replication were successful with similar results.
Randomization	Samples were allocated random, including the particle-motion and structural determination in Relion (version 3.1.3).
Blinding	For cryo-EM reconstruction in Relion (version 3.1.3), particles were randomly divided into two parts, and used for 3D structure determination. The consistence of structures generated by these two sub-datasets was used for the blinding test. The investigators were blinded to group allocation during data collection and/or analysis.

Reporting for specific materials, systems and methods

We require information from authors about some types of materials, experimental systems and methods used in many studies. Here, indicate whether each material, system or method listed is relevant to your study. If you are not sure if a list item applies to your research, read the appropriate section before selecting a response.

Materials & experimental systems

n/a	Involved in the study
☒	☐ Antibodies
☒	☐ Eukaryotic cell lines
☒	☐ Palaeontology and archaeology
☒	☐ Animals and other organisms
☒	☐ Human research participants
☒	☐ Clinical data
☒	☐ Dual use research of concern

Methods

n/a	Involved in the study
☒	☐ ChIP-seq
☒	☐ Flow cytometry
☒	☐ MRI-based neuroimaging
